# Hazards to avoid in future neonatal studies of nasal high-frequency oscillatory ventilation: lessons from an early terminated trial

**DOI:** 10.1186/s13104-019-4268-2

**Published:** 2019-04-25

**Authors:** Hendrik S. Fischer, Christoph Bührer, Christoph Czernik

**Affiliations:** Department of Neonatology, Charité – Universitätsmedizin Berlin, Augustenburger Platz 1, 13353 Berlin, Germany

**Keywords:** Extubation, High-frequency ventilation, Hypercapnia, Nasal continuous positive airway pressure, Premature infant, Randomised controlled trial

## Abstract

**Objective:**

To investigate whether nasal high-frequency oscillatory ventilation (nHFOV) started immediately after extubation of mechanically ventilated very low birth weight infants reduces the partial pressure of carbon dioxide at 72 h after extubation in comparison with nasal continuous positive airway pressure. This randomised controlled single-centre trial aimed to include 68 preterm infants at high risk of extubation failure.

**Results:**

Implementation of the study protocol was feasible. However, from 2015 to 2017, only six patients could be recruited, leading to early termination of the trial. The slow recruitment was due to the introduction of new strategies to avoid endotracheal mechanical ventilation, which reduced the number of eligible infants. Moreover, the included infants failed their extubation more often than anticipated, thereby increasing the required sample size. Based on our single-centre experience, we provide information for study planning and discuss the specific requirements for future trial protocols on nHFOV. The extubation of high-risk infants into nHFOV could well be beneficial, but a multicentric approach is necessary to investigate this hypothesis.

*Trial Registration* Clinicaltrials.gov NCT02340299, on 16 January 2015

**Electronic supplementary material:**

The online version of this article (10.1186/s13104-019-4268-2) contains supplementary material, which is available to authorized users.

## Introduction

Nasal high-frequency oscillatory ventilation (nHFOV) is a promising mode of non-invasive respiratory support used in preterm infants [1, 2]. Notwithstanding, the clinical significance of nHFOV in the treatment of these patients remains uncertain, and high-quality data from randomised controlled trials (RCTs) is limited [3, 4]. Short-term crossover studies and an observational study suggested that nHFOV was more effective than nCPAP in improving the exhalation of CO_2_ [5–7]. To verify this hypothesis, we intended to evaluate whether starting nHFOV immediately after extubation would reduce the partial pressure of carbon dioxide (pCO_2_) at 72 h after extubation in comparison with nasal continuous positive airway pressure (nCPAP) in an RCT of very low birth weight (VLBW) infants < 32 weeks’ gestational age (GA). This RCT started in 2015, but had to be terminated prematurely due to slow patient recruitment.

In the present research note, we reported the limited information available on study feasibility, outcomes and adverse events. More importantly, we aimed to provide information about trial conduct and recruitment efforts, as these information could help future researchers learn from the prior experience of our terminated trial [8].

## Main text

### Methodology

The present single-centre RCT was conducted at the Charité University Medical Centre, Berlin, between 1 January 2015 and 31 December 2017. The study was registered at https://www.clinicaltrials.gov on 16 January 2015 (Identifier NCT02340299).

#### Study population

The study participants were recruited at a tertiary referral centre admitting about 170 VLBW infants annually. Infants at < 32 ^0^/_7_ weeks’ GA with a birth weight < 1500 g were eligible if they had received endotracheal mechanical ventilation for ≥ 120 h, were deemed ready for extubation, and fulfilled the extubation criteria: (1) caffeine treatment according to unit guidelines (2) pCO_2_ < 65 mmHg with a pH > 7.2 (3) fraction of inspired oxygen (F_i_O_2_) of 25–40% to maintain peripheral oxygen saturation (SpO_2_) at 90–94% (4) time-cycled, pressure-controlled ventilation with a peak inspiratory pressure ≤ 22 cm H_2_O and positive end-expiratory pressure (PEEP) ≤ 6 cm H_2_O, volume guarantee ventilation with working peak pressures ≤ 22 cm H_2_O and PEEP ≤ 6 cm H_2_O, or high-frequency oscillatory ventilation with a mean airway pressure (P_mean_) ≤ 12 cm H_2_O and an amplitude ≤ 30 cm H_2_O.

Infants were excluded if they were participants in another RCT, had been treated with hydrocortisone, or exceeded 28 days of chronological age. Other exclusion criteria were major congenital malformation requiring surgery, duct-dependent congenital heart disease, or neuromuscular disease.

#### Randomisation

The eligible infants were randomly assigned to receive nHFOV (intervention group) or nCPAP (control group). An independent statistician and a study nurse performed random sequence generation using a 1:1 ratio and variable block sizes. Allocation concealment was ensured by numbered opaque envelopes opened immediately prior to extubation.

#### Study intervention

After extubation, appropriately sized binasal prongs were applied to the patient (Infant Nasal Prongs, Fisher & Paykel Healthcare Ltd., Auckland, New Zealand) and connected to a heated humidifier (MR850, Fisher & Paykel) and a neonatal ventilator (VN500, Drägerwerk AG, Lübeck, Germany, or Leoni Plus, Heinen & Löwenstein, Bad Ems, Germany) using the manufacturer-approved heated-wire ventilatory circuit. The ventilator remained the same before and after extubation. In the intervention group, nHFOV was provided (frequency 10 Hz; amplitude 20 cm H_2_O; I:E ratio 33:66; P_mean_ 8 cm H_2_O). However, a minimum frequency of 9 Hz, maximum amplitude of 30 cm H_2_O, and maximum P_mean_ of 8 cm H_2_O were applied. In the control group, nCPAP was provided (CPAP level 8 cm H_2_O; flow 7 l/min). The maximum CPAP level was 8 cm H_2_O and the maximum flow 8 l/min. In both groups, the F_i_O_2_ was set to maintain SpO_2_ at 90–94%. Within the limits, the physician was free to adjust ventilator settings.

#### Rescue treatment

Treatment failure of either nHFOV or nCPAP was defined as meeting at least one of the following criteria: (1) a sustained pCO_2_ > 80 mmHg and pH < 7.20 confirmed by arterial or capillary blood gas analysis despite maximum ventilator support (2) an F_i_O_2_ > 0.6 to maintain SpO_2_ at 90–94% (3) reintubation.

If the nCPAP infants developed treatment failure but did not need immediate reintubation, nHFOV was provided as ‘rescue treatment’. In the nHFOV group, any non-invasive rescue treatment could be provided.

#### Primary and secondary outcomes

The primary outcome was the pCO_2_ at 72 h after extubation. The secondary outcomes were pH, partial pressure of oxygen, and base excess at 72 h after extubation, blood gas analysis results at 2 h after extubation, successful extubation within 72 h, airway obstruction due to highly viscous secretions within 72 h, treatment failure within 7 days, reintubation within 7 days, total duration of mechanical ventilation, and total duration of supplemental oxygen until discharge. Other secondary outcomes included rescue treatment and incidences of common adverse effects of prematurity.

#### Sample size calculation, data monitoring, descriptive statistics

Assuming a variability of the pCO_2_, as previously reported for difficult-to-wean preterm infants in our unit [7], and a treatment failure rate of 22% within 72 h after extubation, we calculated a sample size of 34 patients in each study arm to detect a difference in the pCO_2_ of 7 mmHg using a two-sided significance of 0.05 and a power of 0.8. No predefined standards existed for periodical data review or early termination of the trial, and no Data and Safety Monitoring Board (DSMB) was instituted. The categorical data was described in numbers and percentages, the quantitative data as medians and ranges.

### Results

The nHFOV trial was halted on 31 December 2017 due to slow recruitment. Within three years, only six patients had been included in the study.

**Fig. 1:**
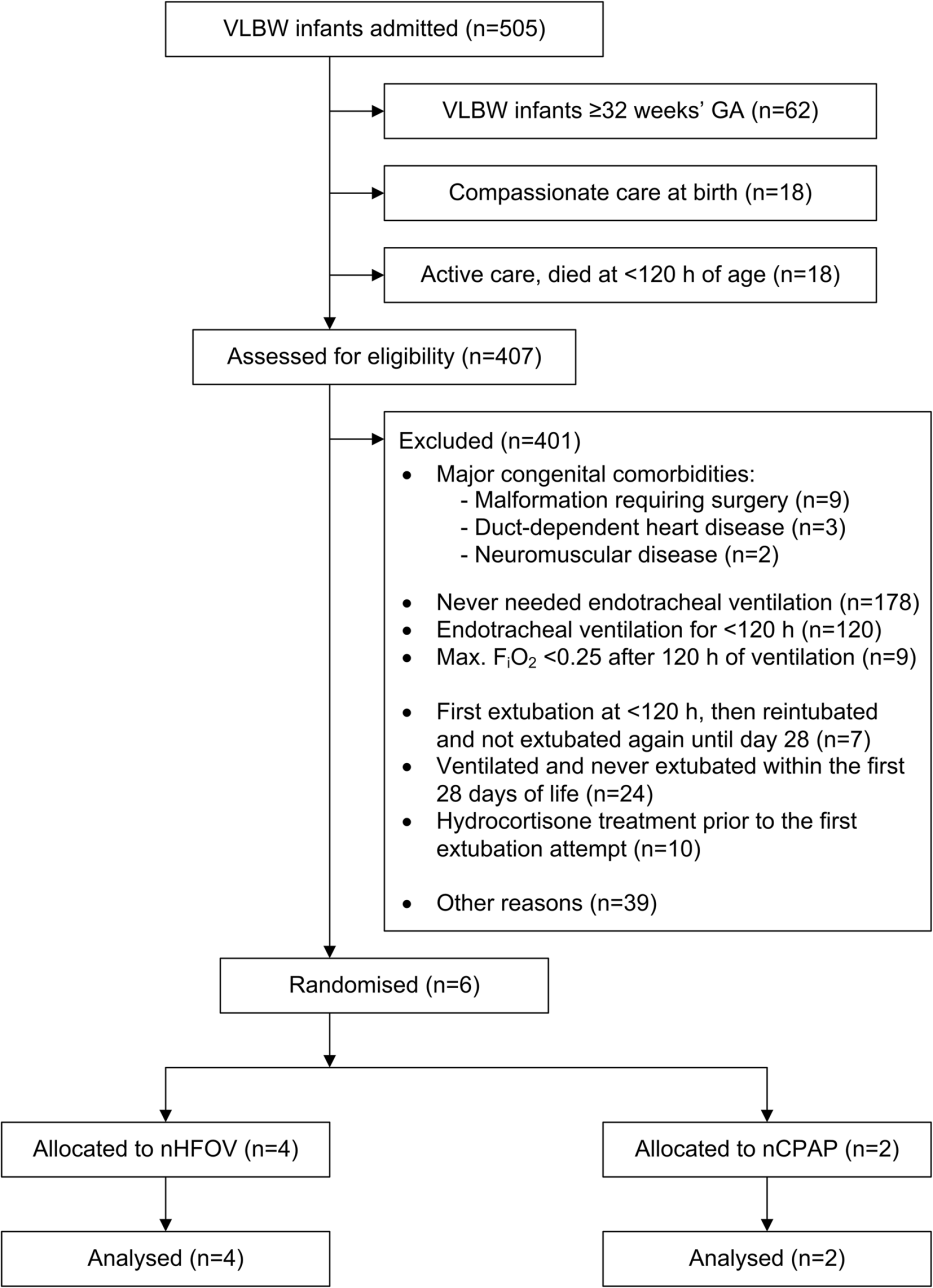
Flow diagram of the progress of the two study groups through the phases of the randomised trial

From January 2015 to December 2017, a total of 407 VLBW infants < 32 weeks’ GA survived to 120 h of age and were assessed for eligibility. The reasons for their exclusion are detailed in Fig. 1. Most infants were not eligible for the study because they had either never been endotracheally ventilated or had been but for < 120 h. Among the ventilated patients, 92% (211/229) received surfactant. Patients excluded due to hydrocortisone treatment had a high risk of mortality and morbidity (see Additional file 1). Notably, 39 infants were not included due to ‘other’ reasons that were mostly not specified in the patients’ records. We are aware that these reasons comprised participation in another RCT, failure to assess study eligibility, failure to approach the parents for consent within the window of opportunity, lack of skilled translators, parental disapproval of study participation, failure to comply with the complex extubation criteria, and accidental extubation.

Table 1 shows the patient characteristics. Two patients suffered from an intraventricular haemorrhage (IVH) 3°–4° prior to randomisation and happened to be randomised to the nCPAP group. All study patients survived to discharge. In the nHFOV group, three out of four patients were successfully extubated, but one required rescue treatment (P_mean_ was increased to 10 cm H_2_O). In the nCPAP group, both patients had to be reintubated within 72 h. The primary outcome, pCO_2_ 72 h after extubation, could therefore only be assessed in the nHFOV group and was 65 (52–79) mmHg. Selected secondary outcomes are shown in Table 2. Apart from the pre-existing IVH 3°–4° cases, there were no obvious differences in any adverse outcomes. The small number of study patients in each group precluded any formal statistical comparison.

**Table 1:** Characteristics of the study patients, median (range) or n (%)

	nHFOV (n = 4)	nCPAP (n = 2)
At birth		
Gestational age (weeks)	25 ^0^/_7_ (23 ^4^/_7_–26 ^3^/_7_)	24 ^0^/_3_ (23 ^6^/_7_–24 ^6^/_7_)
Birth weight (g)	503 (420–568)	668 (550–786)
Male	2 (50)	1 (50)
At study entry		
Chronological age (days)	19 (15–28)	16 (13–19)
Actual weight (g)	667 (475–700)	800 (775–825)
Surfactant treatment	4 (100)	2 (100)
P_mean_ (cm H_2_O)	8.5 (8.3–9.5)	8.2 (6.4–10)
F_i_O_2_ (%)	31 (30–38)	26.5 (25–28)
Intraventricular haemorrhage 3°–4°	0 (0)	2 (100)

**Table 2:** Selected secondary outcomes, median (range) or n (%)

	nHFOV (n = 4)	nCPAP (n = 2)
Blood gas analysis 2 h after extubation		
pH	7.31 (7.20–7.36)	7.25^a^
pCO_2_ (mmHg)	60 (52–63)	73^a^
pO_2_ (mmHg)	32 (25–42)	35^a^
Other secondary outcomes		
Successful extubation for > 72 h	3 (75)	0 (0)
Treatment failure	2 (50)	2 (100)
Successful rescue treatment	1 (25)	0 (0)
Reintubation	1 (25)	2 (100)
Highly viscous secretions	2 (50)	0 (0)
Duration of mechanical ventilation (days)	22.5 (15–54)	47 (40–54)
Duration of supplemental oxygen (days)	70 (51–118)	81 (80–82)
Intraventricular haemorrhage 3°–4°	0 (0)	2 (100)
Death or moderate to severe bronchopulmonary dysplasia^b^	1 (25)	1 (50)

^a^One patient in the nCPAP group had no blood gas analysis due to reintubation less than 2 h after extubation.

^b^Combined outcome of death or moderate to severe bronchopulmonary dysplasia

### Discussion

The present RCT failed due to slow patient recruitment. However, we would like to share the experience of our single-centre trial to facilitate the design of future multicentre RCTs about nHFOV.

#### Feasibility of the trial protocol

Implementation of the study protocol was feasible. The patients included in the trial were at an extremely high risk of re-intubation, with 4/6 (67%) reaching the prespecified criteria of treatment failure and 3/6 (50%) being reintubated. This rate was much higher than anticipated, generated early drop-outs before the primary outcome assessment, and increased the estimated sample size from 34 to 79 patients in each study arm.

#### Impact of nHFOV on study outcomes

Due to the limited number of study patients, a meaningful outcome assessment was not possible.

#### Reasons for slow recruitment

The main reason for slow recruitment was new, clinically beneficial treatment strategies to avoid endotracheal mechanical ventilation, such as less-invasive surfactant application (LISA) [9]. By the start of the nHFOV trial in 2015, LISA had just become our routine treatment for VLBW infants with respiratory distress syndrome. Consequently, less patients fulfilled the study eligibility criteria of receiving ≥ 120 h of endotracheal mechanical ventilation and requiring a F_i_O_2_ of 25–40% at extubation. VLBW infants with complicated clinical courses (e.g. IVH 3°–4°, patent ductus arteriosus, abdominal surgery) still required prolonged ventilation and a high F_i_O_2_. Many of these severely affected patients, however, were not eligible because they had either received hydrocortisone for their first extubation attempt or had not been extubated at any time within the first 28 days of life (Fig. 1). This shows how challenging it is in the era of LISA to target a population of infants at high risk of extubation without including those receiving postnatal steroids. With regard to the infants for whom the reason for exclusion was not clearly documented, we now estimate that maximising our efforts could have increased the overall recruitment to 10–15 patients in the 2015–2017 period. Nevertheless, we would have still required more than 30 years to include 79 patients in each study arm. The RCT was therefore halted prematurely.

#### Requirements for future trial protocols

There are several key elements that should be considered in future RCTs of nHFOV. Most importantly, the inclusion criteria should specifically target infants who might benefit from nHFOV. The beneficial effects of nHFOV may be difficult to show in mildly affected infants already stabilised on nCPAP, which may explain why nHFOV was shown to have had no impact on pCO_2_ in a recent cross-over trial [10]. By contrast, the present pilot RCT showed that targeting high-risk patients would require a multicentric approach.

Second, our experience showed that overly complex study inclusion criteria may interfere with recruitment. Pragmatic criteria, for example, the inclusion of all VLBW infants extubated after postnatal steroid treatment, may facilitate the recruitment of high-risk patients in future RCTs.

Third, there is the question of choosing the most appropriate primary outcome. A rise in pCO_2_ is a reliable indicator of respiratory fatigue in patients with pulmonary failure. Nobody knows, however, whether the beneficial effects of nHFOV on gas exchange are reflected by the pCO_2_ in stable patients as these patients may simply decrease their respiratory efforts to keep the pCO_2_ at the previous level. Work of breathing or the pressure–time product would be more sensitive primary outcomes but are difficult to measure during neonatal non-invasive respiratory support [11]. From a clinical point of view, successful extubation or ventilation/perfusion mismatch and bronchopulmonary dysplasia assessed in a standard oxygen reduction test would be more relevant outcomes [12], but these would require larger sample sizes.

Fourth, future trials should assess potential side effects of nHFOV, such as upper airway obstruction due to highly viscous secretions [1]. Notably, a recent in vitro study showed that this effect may occur due to impaired oropharyngeal gas conditioning in the presence of vigorous nHFOV settings [13].

Fifth, future RCTs should institute clear stopping rules and a DSMB to facilitate decisions regarding the continuation, modification, or termination of the trial.

Finally, it should be mentioned that nHFOV may be beneficial in the primary treatment of respiratory distress syndrome [14], but a multicentre RCT of very preterm infants of 24 to 28 weeks’ GA would be necessary to investigate this in more detail [15]. NHFOV may also be an effective rescue treatment in infants with nCPAP failure, but again, a multicentric approach would be needed to investigate this hypothesis [16].

## Limitations of this RCT

- New treatment strategies to avoid endotracheal ventilation reduced the number of eligible patients.
- Complex inclusion criteria interfered with patient recruitment.
- The treatment failure rate was higher than expected.
- There were no predefined criteria for trial termination and no DSMB.
- The trial was halted prematurely due to slow patient recruitment.

## Additional file

**Additional file 1.** Patient characteristics and outcomes of very low birth weight infants < 32 weeks’ gestational age ventilated for ≥ 120 h, who were excluded from the study due to hydrocortisone treatment (n = 10).

## Abbreviations

DSMB: Data and Safety Monitoring Board
F_i_O_2_: fraction of inspired oxygen
GA: gestational age
IVH: intraventricular haemorrhage
LISA: less-invasive surfactant application
nCPAP: nasal continuous positive airway pressure
nHFOV: nasal high-frequency oscillatory ventilation
pCO_2_: partial pressure of carbon dioxide
PEEP: positive end-expiratory pressure
P_mean_: mean airway pressure
RCT: randomised controlled trial
SpO_2_: peripheral oxygen saturation
VLBW: very low birth weight

## References

[1] Fischer HS, Bohlin K, Bührer C, Schmalisch G, Cremer M, Reiss I, Czernik C (2015). Nasal high-frequency oscillation ventilation in neonates: a survey in five European countries. Eur J Pediatr..

[2] Mukerji A, Shah PS, Shivananda S, Yee W, Read B, Minski J, Alvaro R, Fusch C (2017). Canadian Neonatal Network I: survey of noninvasive respiratory support practices in Canadian neonatal intensive care units. Acta Paediatr..

[3] De Luca D, Dell'Orto V (2016). Non-invasive high-frequency oscillatory ventilation in neonates: review of physiology, biology and clinical data. Arch Dis Child Fetal Neonatal Ed..

[4] Mukerji A, Dunn M (2016). High-frequency ventilation as a mode of noninvasive respiratory support. Clin Perinatol..

[5] Colaizy TT, Younis UM, Bell EF, Klein JM (2008). Nasal high-frequency ventilation for premature infants. Acta Paediatr..

[6] Bottino R, Pontiggia F, Ricci C, Gambacorta A, Paladini A, Chijenas V, Liubsys A, Navikiene J, Pliauckiene A, Mercadante D (2018). Nasal high-frequency oscillatory ventilation and CO_2_ removal: a randomized controlled crossover trial. Pediatr Pulmonol..

[7] Czernik C, Schmalisch G, Bührer C, Proquitte H (2012). Weaning of neonates from mechanical ventilation by use of nasopharyngeal high-frequency oscillatory ventilation: a preliminary study. J Matern Fetal Neonatal Med..

[8] Williams RJ, Tse T, DiPiazza K, Zarin DA (2015). Terminated trials in the clinicaltrials.gov results database: evaluation of availability of primary outcome data and reasons for termination. Plos ONE.

[9] Kribs A, Roll C, Göpel W, Wieg C, Groneck P, Laux R, Teig N, Hoehn T, Bohm W, Welzing L (2015). Nonintubated surfactant application vs conventional therapy in extremely preterm infants: a randomized clinical trial. JAMA Pediatr..

[10] Klotz D, Schneider H, Schumann S, Mayer B, Fuchs H (2018). Non-invasive high-frequency oscillatory ventilation in preterm infants: a randomised controlled cross-over trial. Arch Dis Child Fetal Neonatal Ed..

[11] Montecchia F, Midulla F, Papoff P (2018). A flow-leak correction algorithm for pneumotachographic work-of-breathing measurement during high-flow nasal cannula oxygen therapy. Med Eng Phys..

[12] Bamat N, Ghavam S, Liu Y, DeMauro SB, Jensen EA, Roberts R, Yoder BA, Kirpalani H (2015). Reliability of a noninvasive measure of V/Q mismatch for bronchopulmonary dysplasia. Ann Am Thorac Soc..

[13] Ullrich TL, Czernik C, Bührer C, Schmalisch G, Fischer HS (2017). Nasal high-frequency oscillatory ventilation impairs heated humidification: a neonatal bench study. Pediatr Pulmonol..

[14] Zhu XW, Zhao JN, Tang SF, Yan J, Shi Y (2017). Noninvasive high-frequency oscillatory ventilation versus nasal continuous positive airway pressure in preterm infants with moderate-severe respiratory distress syndrome: a preliminary report. Pediatr Pulmonol..

[15] Fischer HS, Rimensberger PC (2018). Early noninvasive high-frequency oscillatory ventilation in the primary treatment of respiratory distress syndrome. Pediatr Pulmonol..

[16] Mukerji A, Sarmiento K, Lee B, Hassall K, Shah V (2017). Non-invasive high-frequency ventilation versus bi-phasic continuous positive airway pressure (BP-CPAP) following CPAP failure in infants <1250 g: a pilot randomized controlled trial. J Perinatol..

