# Supplementary material for: Hazards to avoid in future neonatal studies of nasal high-frequency oscillatory ventilation: lessons from an early terminated trial
Source: BMC Res Notes. 2019 Apr 25;12:237. doi: 10.1186/s13104-019-4268-2 (PMC6482494; doi:10.1186/s13104-019-4268-2)
Supplement: Supplementary file 1 — Additional file 1. Patient characteristics and outcomes of very low birth weight infants < 32 weeks’ gestational age ventilated for ≥ 120 h, who were excluded from the study due to hydrocortisone treatment (n = 10). [file 13104_2019_4268_MOESM1_ESM.pdf]

Additional file 1: Patient characteristics and outcomes of very low birth weight infants <32 weeks' gestational age ventilated for ≥120h, who were excluded from the study due to hydrocortisone treatment (n=10)

	Median (range) or n (%)
Patient characteristics	
Gestational age [weeks]	24 $^{1}/_{7}$ (22 $^{4}/_{7}$–26 $^{2}/_{7}$)
Birth weight [g]	637.5 (390–1110)
Surfactant	10 (100)
Outcomes	
Intraventricular haemorrhage grade 3–4	2 (20)
Surgical patent ductus arteriosus	2 (20)
Surgical necrotising enterocolitis	0 (0)
Retinopathy of prematurity requiring laser or bevacizumab treatment	2 (20)
Pneumothorax	2 (20)
Periventricular leucomalacia	0 (0)
Bronchopulmonary Dysplasia	8 (80)
Death	2 (20)
Combined outcome of death or moderate to severe bronchopulmonary dysplasia	9 (90)
